# Presence of additional *Plasmodium vivax* malaria in Duffy negative individuals from Southwestern Nigeria

**DOI:** 10.1186/s12936-020-03301-w

**Published:** 2020-06-26

**Authors:** Mary Aigbiremo Oboh, Upasana Shyamsunder Singh, Daouda Ndiaye, Aida Sadikh Badiane, Nazia Anwar Ali, Praveen Kumar Bharti, Aparup Das

**Affiliations:** 1 Medical Research Council Unit The Gambia at LSHTM, Fajara, P.O. Box 273, Banjul, Gambia; 2 School of Earth and Environmental Sciences, University of Manchester, Manchester, UK; 3 Genomic Epidemiology Laboratory, Division of Vector Borne Diseases, ICMR-National Institute of Research in Tribal Health, Jabalpur, India; 4 Parasitology and Mycology Laboratory, Université Cheikh Anta Diop, Dakar, Senegal; 5 National Institute of Research in Tribal Health, Jabalpur, Madhya Pradesh 482003 India

**Keywords:** Sub-Saharan Africa, Duffy Antigen Receptor for Chemokines, *Plasmodium vivax*, Mix-infection, Genetic-epidemiology

## Abstract

**Background:**

Malaria in sub-Saharan Africa (sSA) is thought to be mostly caused by *Plasmodium falciparum*. Recently, growing reports of cases due to *Plasmodium ovale*, *Plasmodium malariae*, and *Plasmodium vivax* have been increasingly observed to play a role in malaria epidemiology in sSA. This in fact is due to the usage of very sensitive diagnostic tools (e.g. PCR), which have highlighted the underestimation of non-falciparum malaria in this sub-region. *Plasmodium vivax* was historically thought to be absent in sSA due to the high prevalence of the Duffy negativity in individuals residing in this sub-continent. Recent studies reporting detection of vivax malaria in Duffy-negative individuals from Mali, Mauritania, Cameroon challenge this notion.

**Methods:**

Following previous report of *P. vivax* in Duffy-negative individuals in Nigeria, samples were further collected and assessed RDT and/or microscopy. Thereafter, malaria positive samples were subjected to conventional PCR method and DNA sequencing to confirm both single/mixed infections as well as the Duffy status of the individuals.

**Results:**

Amplification of *Plasmodium* gDNA was successful in 59.9% (145/242) of the evaluated isolates and as expected *P. falciparum* was the most predominant (91.7%) species identified. Interestingly, four *P. vivax* isolates were identified either as single (3) or mixed (one *P. falciparum*/*P. vivax*) infection. Sequencing results confirmed all vivax isolates as truly vivax malaria and the patient were of Duffy-negative genotype.

**Conclusion:**

Identification of additional vivax isolates among Duffy-negative individuals from Nigeria, substantiate the expanding body of evidence on the ability of *P. vivax* to infect RBCs that do not express the DARC gene. Hence, such genetic-epidemiological study should be conducted at the country level in order to evaluate the true burden of *P. vivax* in Nigeria.

## Background

Malaria is a critical infectious disease of public health importance that provokes considerable mortality in all endemic countries. The tremendous gains seen in cases and mortality reduction is as a result of deliberate intervention strategies [1]. However, the observed benefits have seen a plateau in the last 2 years especially in Africa, where, the greatest burden of disease is mostly impacted. In sub-Saharan Africa (sSA), the majority (99%) of the malaria infections is thought to be due to *Plasmodium falciparum* and, rarely by *Plasmodium ovale*, *Plasmodium malariae*, while *Plasmodium vivax* is not even considered as one of the players [1] in shaping malaria epidemiology in sSA. With the availability of tools that are more sensitive, the detection of non-falciparum and even vivax human malaria parasites has gained more attention in sSA [2–6].

Historically, *P. vivax* prevails in Asia, [7, 8], South America [9, 10] and has some scanty presence in the Horns of Africa, such as in Djibouti [11], Eritrea [12], Somalia [13, 14], Ethiopia [15–18] and Sudan [19, 20]. Thus, *P. vivax* has a much wider geographical distribution outside of Africa unlike falciparum malaria. Hence, the former notion that *P. vivax* originates from Asia and South America, then gradually finds its way into Africa through the trade-route corridor is being opposed by current evidences. These recent proofs support the hypothesis that, *P. vivax* could have originally evolved from a vivax-like strain detected in non-human primates in Africa [21, 22] and, from there dispersed to other continents during the period of human migration. Although, both hypotheses (whether from Africa to Asia or, Asia to Africa) require further validation. Notwithstanding that, it seems likely that there might be an interplay of both hypotheses, in which case, simultaneous occurrence and selective adaptation of the Duffy negative allele in sSA might have resulted in the absence of vivax malaria in the region. Nonetheless, later re-introduction of *P. vivax* into sSA might have happened when individuals expressing the Duffy null allele travels between continents and countries where *P. vivax* is endemic.

The Duffy (gp-*FY;* CD234) gene is the fourth red blood cell (RBC) gene after thalassemia, sickle cell anaemia and glucose-6-phosphate dehydrogenase (G6PD) associated with resistance to *Plasmodium* species [23] albeit with particular protection against vivax malaria. Also known as the Duffy antigen receptor for chemokines (DARC), it is a variable receptor usually expressed on the surface of the red blood cell (RBC) and employed by *P. vivax* merozoites in gaining access in the RBCs and establishing its erythrocytic infection [24]. DARC which is located on chromosome 1 has two exons and a single nucleotide substitution from a thymine (T) to a cytosine (C) upstream of the promoter region that nullifies the expression on RBCs ultimately resulting in the FYO* allele [25]. This FYO* null allele predominates amongst sSA inhabitants as with African-Americans but, has a very sparse representation in individuals of other ancestry [26]. Thus, the FYO* null allele has been validated to confer protection against *P. vivax* infection [23] in this sub-region. Nevertheless, 11 countries in this region (Oboh et al. unpublished) have reported the occurrence of *P. vivax* making it more real that vivax malaria might be gradually finding its way into sSA, and it can, therefore, be postulated that hidden transmission is occurring in this region. In some of these studies, such as in those conducted in Angola, Cameroon, Kenya, Madagascar, Mali and Mauritania, the Duffy status of the infected individuals was characterized and they were found to be mostly Duffy negative [3, 4, 27–30]. In others, however, the investigators were concerned with the identification of *P. vivax* without stating the Duffy status of the infected individuals [5, 31–33]. Interestingly, all studies were carried out amongst indigenous individuals with little or no travel history to other vivax endemic areas. Therefore, the possibility of imported infection can be ruled out.

In Nigeria, *P. falciparum* is responsible for > 95% of malaria infection, with *P. malariae* and *P. ovale* contributing a meagre < 5% of infection [1, 34]. Data implicating *P. vivax* infection in Nigeria include its detection in a visiting pregnant female [35] and, two cases detected by microscopy [36, 37], both of which were not confirmed by any molecular technique. Nevertheless, evidence from previous data molecularly (by PCR) confirmed [6] five Duffy negative individuals to be infected with *P. vivax* isolates from Nigeria. Interestingly, all the vivax malaria cases had been subsequently confirmed by capillary sequencing. Thus, as a follow-up to our previous study, samples were collected from two sites—Oredo and Kosofe in Edo and Lagos state and evaluated with the classical PCR method. In order to confirm these additional *P. vivax* isolates (both single and mixed infection), sequencing by Big Dye Terminator was done. In addition, the Duffy status of the individuals was determined. The importance of such genomic epidemiological studies cannot be underestimated in this era of malaria elimination, as attention also needs to be given to non-falciparum infection, if the ambitious, albeit achievable 2030 elimination goal is to be reached.

## Methods

Ethical approval for this study was obtained from the Institutional review board of the Nigerian Institute of Medical Research and only consenting individuals were enrolled in the study.

Blood samples were purposefully collected from all symptomatic patients attending two hospitals in Lagos (Gbagada) and Edo (Central) states within the study duration (December 2016–January 2017). Patients were recruited if they are ≥ 2 years and, do not have any severe medical conditions, while pregnant women and nursing mothers were excluded from the study. Samples were quickly subjected to malaria rapid diagnostic test kit, employing the manufacturer’s instruction (Pf-HRPII- Care Start^®^, Access Bio Inc, Batch number M014L04-M014M10) followed by microscopy by a World Health Organization (WHO) expert microscopist. Two dried blood-spots (DBS) per patient per filter paper (242 in total), irrespective of their status (positive or negative by any of the techniques above) were made on Whatmann^®^ (GE Healthcare, Life Sciences) filter paper. All DBS were brought to the ICMR-National Institute of Research in Tribal Health (ICMR-NIRTH), Jabalpur, India, where all molecular including DNA sequencing work have been conducted.

Employing the Qiagen^®^ Mini kit (the QIAamp DNA Blood Mini Kit; Hilden, Germany), genomic DNA was isolated from all 242 samples and subsequently subjected to nested PCR diagnostic protocol targeting the 18S rRNA to identify all four *Plasmodium* infecting species using primer pairs as designed earlier [38]. For each PCR run, a negative control (nuclease free water) and positive controls (sequenced confirmed *Plasmodium* species-for all four species) were added. In addition, a part of the promoter region of Duffy gene (for isolates that are *P. vivax* positive) was PCR amplified and sequenced in order to determine their Duffy status using protocols and primer sequence detailed in our previous work [6]. Representative isolates of *Plasmodium* species (*P. falciparum, P. vivax, P. malariae* and *P*. *ovale*) were purified (using Fast^AP^ alkaline phosphatase and exonuclease I) and processed for sequencing by Sanger method (an in-house facility of ICMR-NIRTH, Jabalpur) in both direction (2X coverage). Sequencing was performed on the purified PCR products in a volume of 10 µl with 0.5 µl of Terminator ready reaction mix (TRR), 1.6 pmol of gene specific primer and 5X reaction buffer with a cycling condition of 96 °C denaturation for 10 s (25 cycles), annealing at 50 °C for 5 s and an extension of 60 °C for 4 minutes. Base calling of nucleotide and chromatogram visualization was achieved using the sequence analysis software accompanying the DNA analyser (Sequence analyser™), while sequence alignment was carried out with BioEdit sequence alignment editor v.7.0.5.3. Contiguous sequences were aligned with their respective reference strains (*P. vivax*-SAL-1 accession number U03079.1; *P. falciparum*-3D7 accession umber XR_002273095.1; *P. ovale*-accession number L48987.1; *P. malariae*-accession number NG_011626.30 and, the Duffy gene; accession number NG_011626.30). In addition, the positive predictive value, negative predictive value and Kappa’s test statistics were determined for each diagnostic tool in order to evaluate their performance.

## Results

Between December 2016 and January 2017, a total of two hundred and forty-two samples were collected from both study areas, with majority of the samples (171) being from Oredo in Edo State. The mean age group from both localities are almost the same, 25 years in Kosofe and 26 years in Oredo. As with the mean age, the ratio of male to female is almost same (1:1.2) (Table 1). All 242 samples were subjected to the three diagnostic tools (RDT, microscopy and PCR) and the outcome were widely different. While RDT (187) and PCR (145) gave the highest positive results, that of microscopy was abysmally poor detecting only 53 positive isolates. Thus, using PCR as the gold standard RDT gave a higher sensitivity (84.8%), although with a low specificity (34%). On the other hand, the specificity of microscopy was remarkably higher (82.5%) than what was obtained by RDT.

**Table 1:** Background information of the study participants

	Kosofe	Oredo	Total
Number	71	171	242
Percentage (%)	29.3	70.7	100
Age (years)			
Mean	25	26	
Range	2–85	2–86	
Sex			
Male	36	73	109
Female	35	98	133

A converse pattern was noticed with regards to the likelihood of a positive or negative sample being correctly identified as such. For RDT, the chances of a positive samples turning out positive by PCR-positive predictive value (PPV) was lower (65.8%) than what was observed with microscopy (67.9%) while the chances of it being picked as truly negative; negative predictive value (NPV) was high (60%)(Table 2).

**Table 2:** Diagnostic performance of the different tools

	PCR		Sensitivity (%)	Specificity (%)	PPV (%)	NPV (%)	Kappa’s test	*P* value
	Positive	Negative						
Microscopy								
Positive	36	17	24.8	82.5	67.9	42.3	0.063	0.18
Negative	109	80						
RDT								
Positive	123	64	84.8	34.0	65.8	60.0	0.203	0.01
Negative	22	33						

**Fig. 1:**
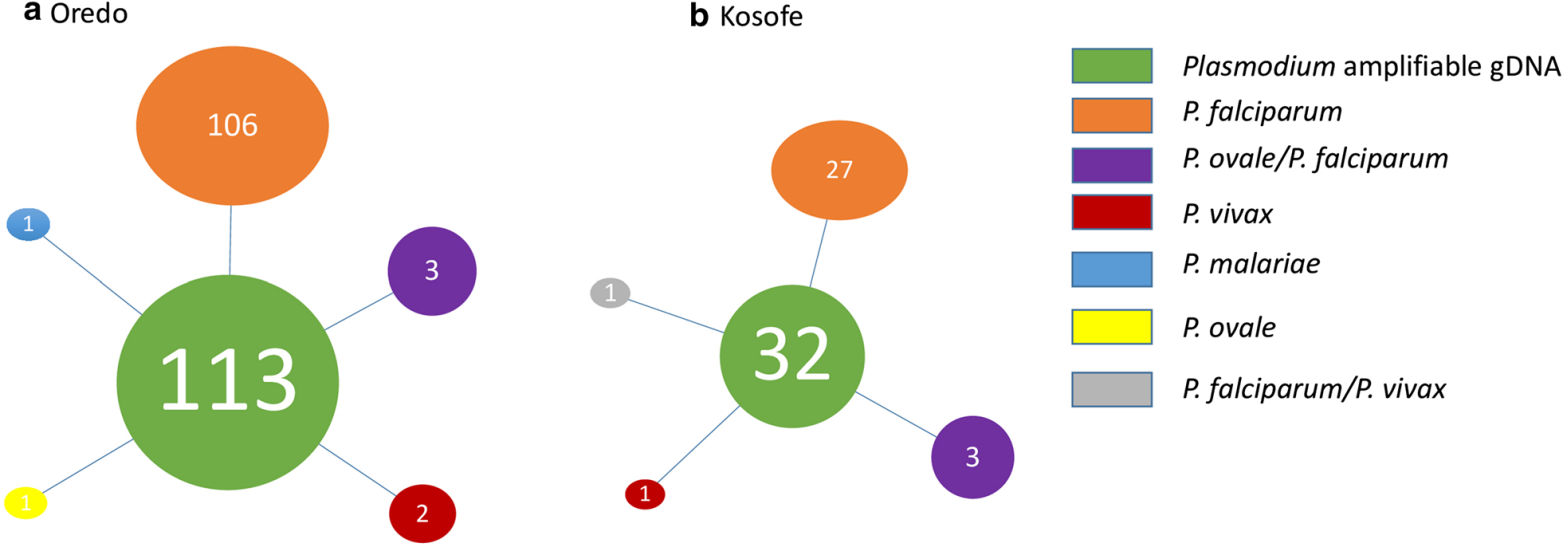
Proportional dynamics of *Plasmodium* species in both study locations

**Fig. 2:**
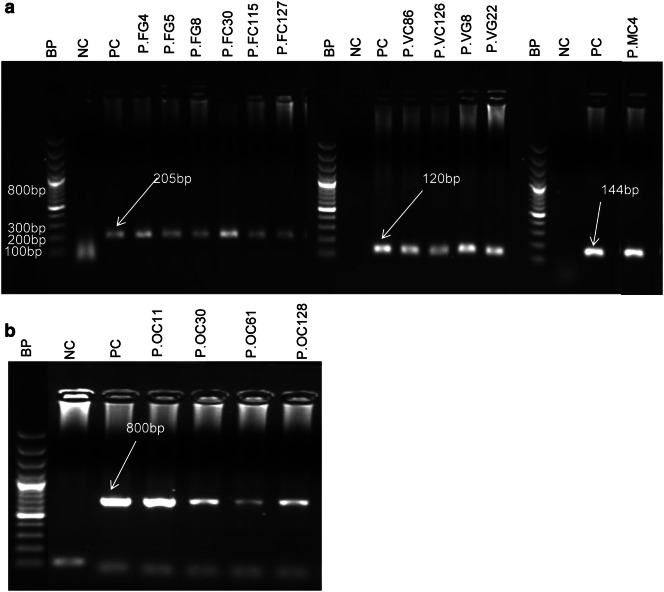
Gel documentation of various *Plasmodium* species. **a** First well—DNA base pair ladder (100 bp), well 2: NC- negative control template (distill water), well 3- PC-P*. falciparum* positive control, well 4-9- isolates of *P. falciparum*, well 10- DNA base pair ladder, wells 11 and 12- negative and positive controls of *P. vivax*, wells 13-16- *P. vivax* samples, well 17- DNA base pair ladder (100 bp), 18 and 19- negative and positive controls of *P. malariae*, well 20- the only additional *P. malariae* detected. **b** well 1- DNA base pair ladder (100 bp), wells 2 and 3- negative and positive controls of *P. ovale*, wells 4-7- *P. ovale* isolates

As expected, *P. falciparum* was the most abundant malaria species detected in both localities (106 in Oredo and 27 in Kosofe). The occurrence of other species in both states were rare either in single (one *P. malariae*, one *P. ovale*, three *P. vivax*) or mixed infections (six *P. ovale*/*P. falciparum*, one *P. falciparum*/*P. vivax* –1) (Fig. 1). The gDNA of the identified *P. vivax* isolates were re-extracted and amplified twice following a protocol described earlier [6] in order to be sure of their status. Gel documentation of all newly identified isolates is presented in Fig. 2.

**Fig. 3:**
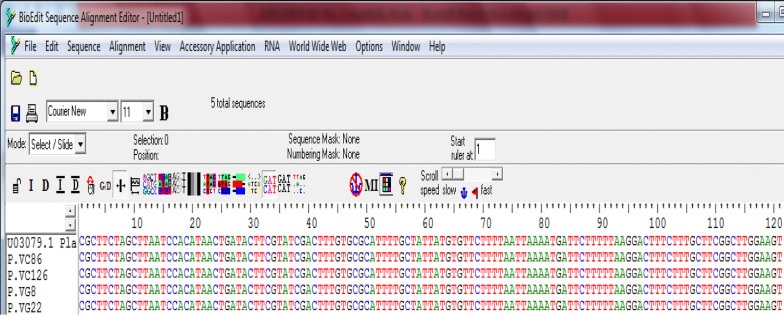
Multiple sequence alignment of *P. vivax* isolates after clean-up and trimming with its Sal -1 reference sequence

Surprisingly, all *P. vivax* isolates sequences showed perfect homology (100% similarity) with their references (Fig. 3 for *P. vivax*).

**Fig. 4:**
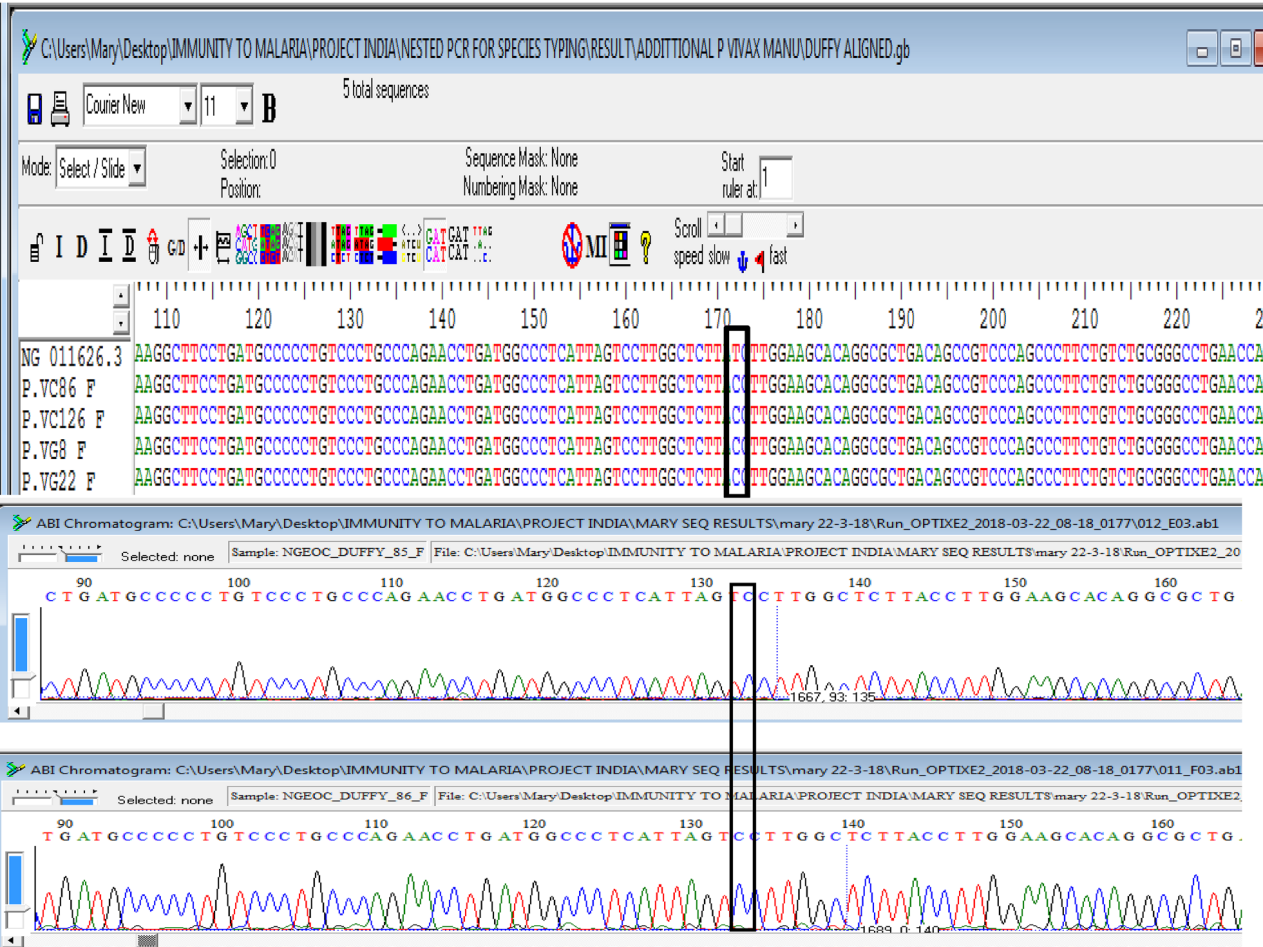
Multiple sequence alignment of the Duffy gene of the vivax samples displaying the—T33C nucleotide change which validates their Duffy negative status

In order to discern the Duffy status of those *P. vivax* infected patients, a portion of the DARC gene (precisely the promoter region covering the T33C point mutation) was amplified and sequenced following previous protocol [39]. Unanticipatedly, all four patients infected with *P. vivax* carried a single cytosine (C) peak at the 33rd nucleotide position upstream (Fig. 4), confirming that none of them expressed the Duffy gene on their RBCs and as such are Duffy negative.

## Discussion

*Plasmodium* genomic DNA was amplifiable in more than half of the isolates. Non-amplification of other samples including those positive by microscopy could be due to a number of reasons from low parasite concentration, mutation in the annealing sites of the target gene to PCR technical inhibition. The identification of more *P. vivax* isolates among these Duffy-negative individuals from Nigeria substantiate the expanding body of evidence of the ability of *P. vivax* to infect RBCs that do not express the DARC gene. Although, a very recent finding points to another domain on the reticulocyte-transferring receptor 1 as a specific *P. vivax* receptor [40] on the reticulocyte. This is being proposed to be an alternate route of entry by *P. vivax* into the RBCs, however, there is need for further verification. The above hypothesis is one of the proposition being made to support the observation of *P. vivax* in sSA [41]. Another which relies on the first conjecture (assuming it is agreed that *P. vivax* at least possesses alternative invasion pathway), is that the Duffy positive carriers in northern part of Africa and the Afro-Asiatic populations of Sudan, Somalia [20, 42] and Ethiopia [43, 44] serve as reservoir to effect transmission to Duffy negative individuals in those areas as well as other countries (in sSA) through migration. Albeit, this particular hypothesis at play here is yet to be determined. One thing is clear here, the true epidemiological situation of *P. vivax* in sSA in particular and Africa in general is yet to be ascertained.

The detection of additional vivax malaria is not unexpected as cases of *P. vivax* have been identified in many countries in sSA [3–5, 29, 33, 45–48] including Nigeria [6], where, it was thought to be absent due to the non-expression of the DARC gene on the RBC of majority of the population. Thus, this is adding to the growing evidence of the proposed gradual incursion of *P. vivax* into sSA sub-region.

Although this study is limited in its samples size and location, the results give support on the need to re-evaluate the spread of *P. vivax* in sSA. Therefore, it is pertinent to carry out more genetic-epidemiological studies in other areas (for example this study covers only two states out of the thirty-six in Nigeria) of the country as with other sSA countries. This will aid in placing appropriate control strategies to combat the menace of malaria infection in this most affected population and may prevent further spread of *P. vivax* in Africa.

## Data Availability

The datasets supporting the conclusion of this article is available in Genbank (MT515456-MT515459 and MT550678-MT550681). All other data can be made available on request to the authors

## Abbreviations

sSA: sub-Saharan Africa
PCR: Polymerase Chain Reaction
DARC: Duffy Antigen Receptor for Chemokines
RBC: Red blood cell
G6PD: Glucose-6-Phosphate Dehydrogenase Deficiency
TRR: Terminator ready reaction mix
RDT: Rapid diagnostic test
PPV: Positive predictive value
NPV: Negative predictive value
